# Functional optical probing of the hippocampal trisynaptic circuit *in vitro*: network dynamics, filter properties, and polysynaptic induction of CA1 LTP

**DOI:** 10.3389/fnins.2015.00160

**Published:** 2015-05-06

**Authors:** Jens Stepan, Julien Dine, Matthias Eder

**Affiliations:** Department Stress Neurobiology and Neurogenetics, Max Planck Institute of PsychiatryMunich, Germany

**Keywords:** hippocampus, trisynaptic circuit, neuronal network dynamics, filter, theta, voltage-sensitive dye imaging, CA1 LTP

## Abstract

Decades of brain research have identified various parallel loops linking the hippocampus with neocortical areas, enabling the acquisition of spatial and episodic memories. Especially the hippocampal trisynaptic circuit [entorhinal cortex layer II → dentate gyrus (DG) → cornu ammonis (CA)-3 → CA1] was studied in great detail because of its seemingly simple connectivity and characteristic structures that are experimentally well accessible. While numerous researchers focused on functional aspects, obtained from a limited number of cells in distinct hippocampal subregions, little is known about the neuronal network dynamics which drive information across multiple synapses for subsequent long-term storage. Fast voltage-sensitive dye imaging *in vitro* allows real-time recording of activity patterns in large/meso-scale neuronal networks with high spatial resolution. In this way, we recently found that entorhinal theta-frequency input to the DG most effectively passes filter mechanisms of the trisynaptic circuit network, generating activity waves which propagate across the entire DG-CA axis. These “trisynaptic circuit waves” involve high-frequency firing of CA3 pyramidal neurons, leading to a rapid induction of classical NMDA receptor-dependent **long-term potentiation (LTP)** at CA3-CA1 synapses (CA1 LTP). CA1 LTP has been substantially evidenced to be essential for some forms of explicit learning in mammals. Here, we review data with particular reference to whole network-level approaches, illustrating how activity propagation can take place within the trisynaptic circuit to drive formation of CA1 LTP.

## Introduction

Accumulating evidence points to a major role of the **hippocampal formation** in the acquisition and recall of episodic and spatial memories in mammals (Whitlock et al., 2006; van Strien et al., 2009). The idea to investigate the underlying **neuronal network dynamics** is well illustrated by common theories about memory acquisition and consolidation (Frankland and Bontempi, 2005; Teyler and Rudy, 2007). These models incorporate the intrinsic organization of the hippocampal formation, its physiology, and its connectivity with other brain regions to give an account of how information about a relevant experience is stored. Above all, this process involves the repeated activation of hippocampal circuits by specific features of the original experience. Due to reciprocal and hierarchically organized connections between the hippocampal formation and adjacent association areas, the information, which is initially “buffered” in the hippocampus (DG + CA + subiculum), can be transferred to the neocortex for subsequent long-term storage (Teyler and Rudy, 2007; Nakashiba et al., 2008).

KEY CONCEPT 1 **Long-term potentiation (LTP)**

Long-lasting activity-dependent enhancement in transmission strength at chemical synapses. At many central synapses, LTP requires activation of NMDA receptors. LTP is widely thought to be one of the major cellular mechanisms underlying learning and memory.

KEY CONCEPT 2 **hippocampal formation**

Compound brain structure consisting of the dentate gyrus, hippocampus proper (cornu ammonis), subiculum, presubiculum, parasubiculum, and entorhinal cortex. The hippocampal formation is critically involved in several cognitive functions like formation of spatial and episodic memories and spatial navigation.

KEY CONCEPT 3 **neuronal network dynamics**

Complex spatio-temporal patterns of electrical activity in large/meso-scale neuronal networks.

Contemporary neuroscience offers a rich toolbox to probe neuronal functioning, ranging from single-cell and local field potential recording to more indirect analyses of whole brain activity by functional magnetic resonance tomography (Andersen et al., 2007; deCharms, 2008). However, to study the integration of thousands of neurons during information processing, these approaches are limited either by their spatial scale, since recordings from individual or a few neurons (“microscale”) typically provide little information about the associated network, or by the fact that non-invasive imaging methods (“macroscale”) measure a surrogate signal whose spatial and temporal resolution are subject to both physical and biological constraints (Logothetis, 2008; Lewis and Lazar, 2013). Therefore, it is crucial to also engage circuit-centered approaches, which operate at the interface of the aforementioned methods (Buzsaki, 2004; Karayiorgou et al., 2012; Lewis and Lazar, 2013). To directly study how neurons communicate across several synapses in large/meso-scale networks, high-speed optical techniques like **voltage-sensitive dye imaging (VSDI)** proved themselves to be instrumental in covering the methodologically demanding “mesoscale” of neuroscience research (Iijima et al., 1996; Airan et al., 2007b; Refojo et al., 2011; von Wolff et al., 2011; Stepan et al., 2012; Avrabos et al., 2013). VSDI allows the analysis of neuronal activity on a millisecond scale, with micrometer-range spatial resolution, and a scope that spans the entire brain circuits under investigation (Tominaga et al., 2000; Grinvald and Hildesheim, 2004; Airan et al., 2007b; Carlson and Coulter, 2008; Chemla and Chavane, 2010; Stepan et al., 2012).

KEY CONCEPT 4 **voltage-sensitive dye imaging**

Imaging technique that uses fluorescent dyes which stably insert into cytoplasmic membranes and report changes in membrane potential by changes in fluorescence emission.

The hippocampal formation is a brain module that is often mentioned in conjunction with **CA1 LTP**. Reasons for this are that CA1 LTP is heavily used as an experimental model for examining cellular underpinnings of learning and memory, demonstrations that CA1 LTP also naturally occurs in the brain, and strong evidence that CA1 LTP is required for some forms of explicit learning in mammals (Morris et al., 1986; Zola-Morgan et al., 1986; Bliss and Collingridge, 1993; Tsien et al., 1996; Burgess et al., 2002; Malinow, 2003; Gruart et al., 2006; Whitlock et al., 2006; Henneberger et al., 2010).

KEY CONCEPT 5 **CA1 LTP**

LTP at hippocampal CA3-CA1 synapses. CA1 LTP depends on NMDA receptor activation, is normally induced by high-frequency (100 Hz) or theta-burst stimulation of CA3-CA1 projections, possesses the properties of “cooperativity,” “associativity,” and “input-specificity,” and predominantly results from a recruitment of additional AMPA receptors to the postsynaptic membrane.

Much of the knowledge about CA1 activation and induction of CA1 LTP comes from *in vivo* and *in vitro* studies using local field potential or single-cell recording in area CA1, excluding the detection of neuronal activity in upstream regions (Andersen et al., 1966; Herreras et al., 1987; Bliss and Collingridge, 1993; Whitlock et al., 2006). Yet, the well-defined regular structure and, at some locations, unidirectional circuitry (Amaral and Witter, 1989) makes the hippocampal formation an ideal candidate for network-level investigations. The entorhinal cortex (EC) represents the main input/output partner of the hippocampus (Witter et al., 2000), thus creating entorhinal-hippocampal loops, perfectly suited for *in vitro* high-speed imaging studies examining the mechanisms of polysynaptic activity flow and induction of long-term synaptic plasticity (Andersen et al., 1966; Herreras et al., 1987; Buzsáki, 1988; Iijima et al., 1996; Nakagami et al., 1997; Stepan et al., 2012). Here, we review anatomical and functional characteristics of the hippocampal trisynaptic circuit and parallel pathways (e.g., temporoammonic pathway), which constitute the foundation for complex neuronal network dynamics during information processing. Including previous work and our recent findings (Stepan et al., 2012), we describe properties of local circuits in the DG and area CA3 and their interaction to enable activity propagation across several synapses for induction of CA1 LTP. We also discuss how our experimental findings can be integrated in the existing literature and how extensions of VSDI toward an “all optical” approach (e.g., by a combination with optogenetic tools) might prove useful for resolving the neuronal network dynamics underlying higher order brain functions.

## Structural architecture of the hippocampal trisynaptic circuit and parallel pathways

The well-established role of the hippocampus in cognitive processes like memory formation relies, among other things, on remarkable anatomical features. In contrast to the reciprocal connectivity of most other cortical structures, the hippocampus is characterized by a largely unidirectional passage of information through its circuitry. However, before polymodal sensory information enters the hippocampus, it has to pass a hierarchically organized neocortical network. Upon sensory receptor stimulation, primary sensory cortices are the first to become activated, followed by supplemental sensory areas and high-order association cortices. Accordingly, highly processed sensory information is subsequently fed into the EC, with a particular focus on superficial layers II and III (Andersen et al., 2007; Teyler and Rudy, 2007).

The EC is often regarded as the first station of information processing in the hippocampal formation. This notion originates from the observation that its superficial layers provide the main cortical input to the hippocampus, while its deep layers represent the main target of information that returns back from area CA1 and the subiculum. EC layer IV and V neurons in turn project to superficial layers or high-order association cortices. In particular, layer II neurons send their axons via the perforant path to the DG and areas CA3 and CA2. The second major input emerges from layer III neurons, which project via the temporoammonic pathway to the CA1 subfield and the subiculum (Witter et al., 2000; van Strien et al., 2009; Kohara et al., 2014). Moreover, some hippocampal regions are connected to subcortical structures, including the amygdala, the hypothalamus, the medial septum, the raphe nucleus, and the locus coeruleus, completed by a pronounced commissural input from the contralateral hippocampus and an ipsilateral associational loop (Nicoll and Schmitz, 2005; Andersen et al., 2007). Interestingly, activation of the locus coeruleus can induce β-adrenergic receptor-dependent LTP at perforant path-DG synapses (Walling and Harley, 2004).

The famous neuroanatomists Ramón y Cajal and Lorente de No were already attracted by the extremely dense division of the perforant path connecting EC layer II cells with the DG (Lorente de No, 1934; Ramón y Cajal, 1995). These axons provide excitatory synaptic input (in the following abbreviated “EC/DG-input”) on apical dendrites of DG granule cells, which give rise to mossy fibers, the most prominent non-commissural/associational excitatory innervation of CA3 pyramidal neurons. These cells in turn synapse via the glutamatergic Schaffer collaterals onto ipsilateral CA1 pyramidal neurons, thereby completing the hippocampal trisynaptic circuit (Amaral and Witter, 1989). Its prominent anatomical appearance and well-established role in information processing strongly suggest that the trisynaptic circuit is the main route of activity flow through the hippocampus (Nicoll and Schmitz, 2005; Nakashiba et al., 2008; Neves et al., 2008; Daumas et al., 2009).

Recent research points to an additional trisynaptic circuit, which might operate independently from the classical one. In this circuit, a subpopulation of DG granule cells target CA2 pyramidal neurons, which synapse on CA1 counterparts. Although uncertainties remain with regards to the specific role of this pathway and its interaction with the classical trisynaptic circuit, its proposed function is to prevent neuronal hyperactivity by triggering feedforward inhibition of CA3 pyramidal neurons (Kohara et al., 2014).

**Figure 1:**
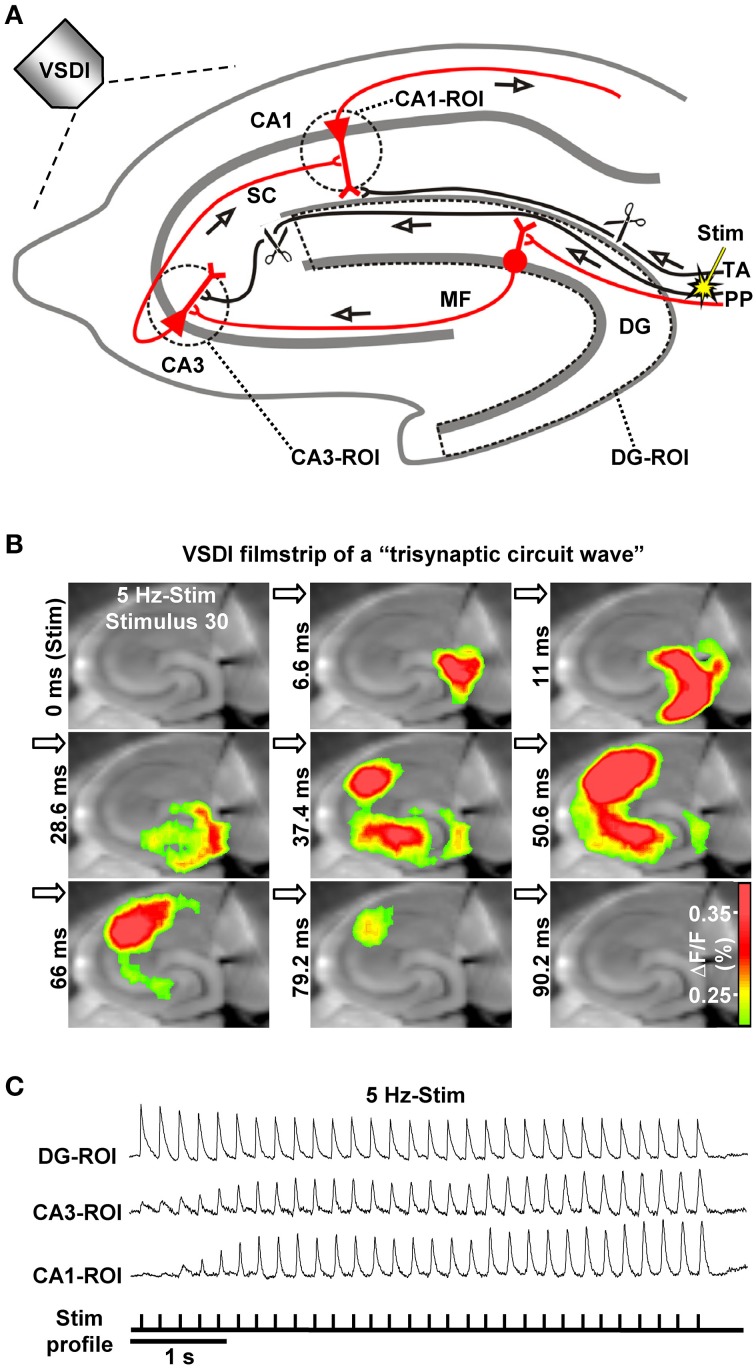
**The hippocampal trisynaptic circuit and monitoring of “trisynaptic circuit waves” by VSDI. (A)** Schematic drawing of the hippocampal trisynaptic circuit (marked in red) and experimental arrangement used for the investigations shown in **(B**,**C**). Scissors illustrate specific deafferentations. **(B,C)** VSDI filmstrip and recording traces depicting trisynaptic circuit waves evoked by theta-frequency (5 Hz) stimulation of perforant path fibers. Warmer colors in **(B)** indicate higher neuronal activity (i.e., excitatory postsynaptic potentials and action potentials; adapted from, Stepan et al., 2012). Abbreviations: ΔF/F, fractional change in fluorescence; MF, mossy fiber; PP, perforant path; ROI, region of interest; SC, Schaffer collateral; Stim, electrical stimulation; TA, temporoammonic pathway.

Together, from the gross anatomy of the hippocampal formation and associated structures, there emerges a complex neuronal network containing several parallel pathways. The trisynaptic circuit represents the most prominent one and is comprised of three excitatory (glutamatergic) synapses (EC layer II → DG → CA3 → CA1, Figure 1A). However, the trisynaptic circuit network does not only consist of trisynaptic interconnections, but also includes associational loops and partly complex interneuronal circuits which mediate feedforward and/or feedback inhibition of principal neurons and disinhibition processes (Andersen et al., 2007; Bartos et al., 2007; Savanthrapadian et al., 2014). Further complicating the picture, several back-projections (e.g., from CA3 pyramidal neurons to the dentate hilus or inner molecular layer of the DG) have been identified (van Strien et al., 2009).

## Voltage-sensitive dye imaging: toward a comprehensive understanding of hippocampal network dynamics

To accurately characterize the activity dynamics and physiological function(s) of a particular hippocampal pathway, several groups deactivated specific fiber tracts *in vivo* or *in vitro* (Remondes and Schuman, 2002; Ang et al., 2005; Hunsaker et al., 2007; Nakashiba et al., 2008; Daumas et al., 2009; Suh et al., 2011; Stepan et al., 2012). *In vivo* investigations allow a direct correlation of manipulations of connectivity patterns with behavioral changes (Hunsaker et al., 2007; Nakashiba et al., 2008; Daumas et al., 2009; Suh et al., 2011). *In vitro* approaches, in which structures like the hippocampus are studied in brain slices, are equally important, since they permit a more precise control of neurophysiological processes (e.g., Butler and Paulsen, 2014). Together, *in vivo* and *in vitro* experiments revealed that each hippocampal pathway alone, but also their interactions, are necessary for normal hippocampal functioning (Remondes and Schuman, 2002; Ang et al., 2005; Nakashiba et al., 2008; Stepan et al., 2012; Kallarackal et al., 2013).

To overcome the limited spatial scale of single-cell and local field potential recordings, several recent studies applied VSDI to hippocampal slice preparations. Most of these investigations examined neuronal network activity in distinct regions of the hippocampal formation like the EC (Canto et al., 2012), the DG (Jackson and Scharfman, 1996; Airan et al., 2007b; Ikrar et al., 2013; Yu et al., 2013; Wright and Jackson, 2014), and area CA1 (Ang et al., 2005; Airan et al., 2007b; Suh et al., 2011; Kim et al., 2012; Dine et al., 2013; Haettig et al., 2013). However, VSDI studies on neuronal activity propagation through the entire trisynaptic circuit are scarce (Iijima et al., 1996; Nakagami et al., 1997; Stepan et al., 2012) and were mostly performed without deactivation of potentially interfering pathways (Iijima et al., 1996; Nakagami et al., 1997).

## Activity dynamics in the trisynaptic circuit network and a putative filter function

Different stimulation paradigms, with varying physiological relevance, have been used *in vivo* and *in vitro* to trigger action potential firing of EC layer II cells for activation of the DG. What emerged from these studies is that low-frequency EC/DG-input (≤0.2 Hz) leads to pronounced neuronal activity in the DG, but only feeble or no activation of CA areas (Herreras et al., 1987; Scharfman, 1991; Stepan et al., 2012; Yu et al., 2013). In contrast, EC/DG-input at 1–20 Hz generates waves of neuronal activity which propagate through the entire trisynaptic circuit network (Herreras et al., 1987; Stepan et al., 2012). These “trisynaptic circuit waves,” which can be accurately monitored by VSDI (Figures 1A,B) and are applicable for pharmacological investigations, start to appear in an initially progressive manner a few hundred milliseconds after the onset of EC/DG-input and precisely follow the input rhythm (Figure 1C; Supplementary Video in Stepan et al., 2012). Furthermore, trisynaptic circuit waves dissipate within a couple of seconds if the inducing EC/DG-input is followed by a low-frequency (e.g., 0.05 Hz) one (Stepan et al., 2012). These findings indicate that the occurrence and strength of activity propagations through the trisynaptic circuit network critically depend on the frequency and persistence of EC/DG-input, a connection which presumably reflects a basic filter mechanism of the hippocampus regarding EC inputs.

## Does the DG-CA3 complex act as a “band-pass filter”?

**Figure 2:**
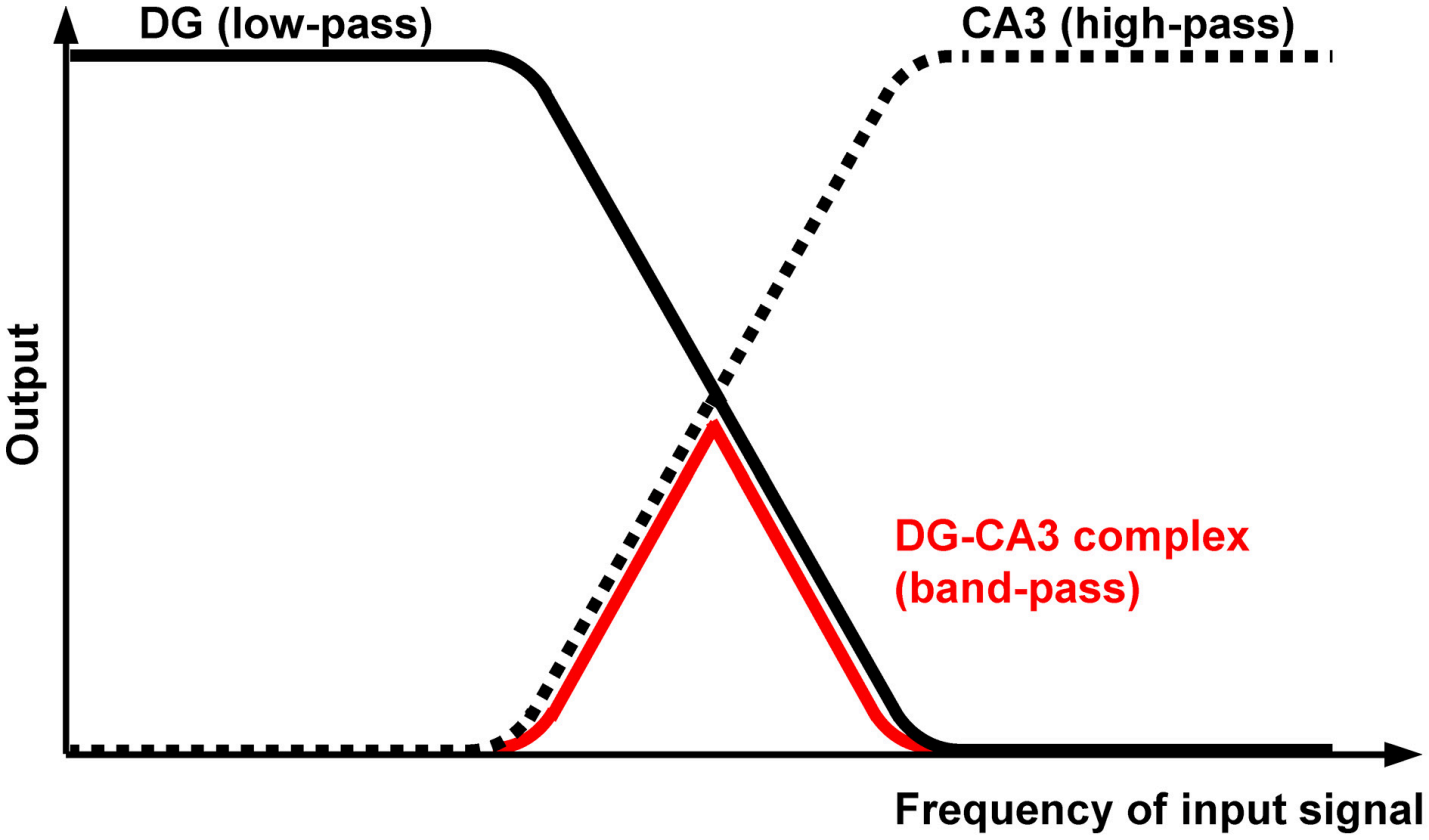
**Proposed filter properties of the DG-CA3 complex**. The DG network operates as a kind of “low-order low-pass filter” (black curve), whereas the CA3 mossy fiber system possesses opposing filter characteristics (dotted curve). The output attenuation ramps overlap, leading to “band-pass” properties of the DG-CA3 complex (red curve) regarding neuronal activity propagation from the EC to area CA1.

A key finding of our previous work is that theta-frequency (5 Hz) EC/DG-input is very effective at generating trisynaptic circuit waves, whereas 1 and 20 Hz EC/DG-input evokes weaker ones. Moreover, we revealed that trisynaptic circuit waves critically depend on frequency facilitation of mossy fiber synaptic transmission onto CA3 pyramidal neurons and observed that DG activity increasingly declines during 0.2, 1, 5, and 20 Hz EC/DG-input, respectively (Stepan et al., 2012). Together with the fact that frequency facilitation at **mossy fiber synapses** develops stronger with higher frequencies (Toth et al., 2000), these results suggest that, regarding neuronal activity propagation from the EC to area CA1, the DG-CA3 complex operates as a kind of “low-order band-pass filter,” wherein the DG network serves as the “low-pass unit” and the CA3 mossy fiber system as the “high-pass device” (**Neural band-pass filter**; Figure 2). If so, this filter would be effectually passed by sensory information encoded in the theta-frequency range. Synchronous theta-rhythmical spiking in EC layer II cell ensembles, as mimicked by our 5 Hz stimulation paradigm (Figure 1), occurs during EC **theta** oscillations, which in turn are tightly linked to several cognitive functions in mammals (Mizuseki et al., 2009; Quilichini et al., 2010; Burgalossi et al., 2011; Colgin, 2013). Given that trisynaptic circuit waves elicit action potentials in pyramidal neurons of the CA1 output subfield of the hippocampus (Herreras et al., 1987; Stepan et al., 2012), an important physiological function of EC theta oscillations thus might be to drive sensory information through the whole entorhinal-hippocampal loop formed by the trisynaptic circuit network.

KEY CONCEPT 6 **Mossy fiber synapse**

Excitatory (glutamatergic) synapse formed by dentate gyrus granule cell axons and CA3 neurons. Mossy fiber synaptic transmission onto CA3 pyramidal neurons exhibits prominent frequency facilitation, a form of presynaptic short-term plasticity which is strongly pronounced in the theta-frequency range and can potentiate the otherwise weak neurotransmission by up to ~1200%.

KEY CONCEPT 7 **“Neural band-pass filter”**

Neural substrate which only generates significant output in response to an input signal if the input signal ranges in a certain frequency band. If the filter displays smooth output attenuation characteristics, it represents a “low-order band-pass filter.”

KEY CONCEPT 8 **Theta**

Frequency band covering ~4–12 Hz of rhythmical (oscillatory) neural activity. Theta activity occurs in various brain structures, including the hippocampal formation, and is tightly linked to mechanisms of learning and memory.

## Putative cellular mechanisms underlying DG and CA3 filter properties

The remarks given above are in line with previous work proposing that the DG acts as a filter to prevent the hippocampus from sensory overflow or runaway excitation as seen in pathological brain states like schizophrenia or temporal lobe epilepsy, respectively (Javanbakht, 2006; Hsu, 2007; Coulter et al., 2011). A filter function was also attributed to the CA3 circuitry (Mori et al., 2004; Zalay and Bardakjian, 2006). But what might be the cellular mechanisms underlying these filter properties?

For area CA3, the situation appears relatively simple. This is because frequency facilitation at mossy fiber synapses onto CA3 pyramidal neurons, which starts to occur at 0.5–1 Hz, develops stronger with higher frequencies and can be so pronounced that even a unitary excitatory postsynaptic potential (EPSP) becomes forceful enough to fire the target cell (Jonas et al., 1993; Toth et al., 2000). Frequency-dependent depression at mossy fiber synapses onto CA3 interneurons, which mediate feedforward inhibition of CA3 pyramidal cells, additionally promotes spiking of these principal neurons (Toth et al., 2000; Mori et al., 2004). Therefore, the CA3 mossy fiber system seems ideally suited to act as a kind of “low-order high-pass filter.”

Although the situation in the DG is presumably more complicated (Hsu, 2007), several findings point to “low-order low-pass filter” properties. For instance, EPSPs at medial perforant path-DG granule cell synapses show frequency-dependent depression, which strongly increases with higher frequencies (Kilbride et al., 2001). Furthermore, a recent study revealed that voltage attenuation in apical dendrites of DG granule cells becomes more severe if the experimentally induced voltage deflections are enhanced in their frequency (Krueppel et al., 2011). A role of inhibitory interneurons must also be taken into account, since repetitive stimulation of perforant path fibers causes habituation of DG granule cell activity, a phenomenon which likewise develops stronger with higher frequencies and involves activation of postsynaptic GABA_B_ receptors (Teyler and Alger, 1976; Rausche et al., 1989). Paired-pulse facilitation at perforant path synapses onto basket cells (Savanthrapadian et al., 2014), which effectively inhibit DG granule cells (Bartos et al., 2007), might additionally contribute to “low-pass filter” properties. Finally, representing another possible mechanism, GABAergic dendritic inhibition of DG granule cells has been found to be more powerful during periods of intense perforant path activity (Liu et al., 2014).

## Trisynaptic induction of CA1 LTP

**Figure 3:**
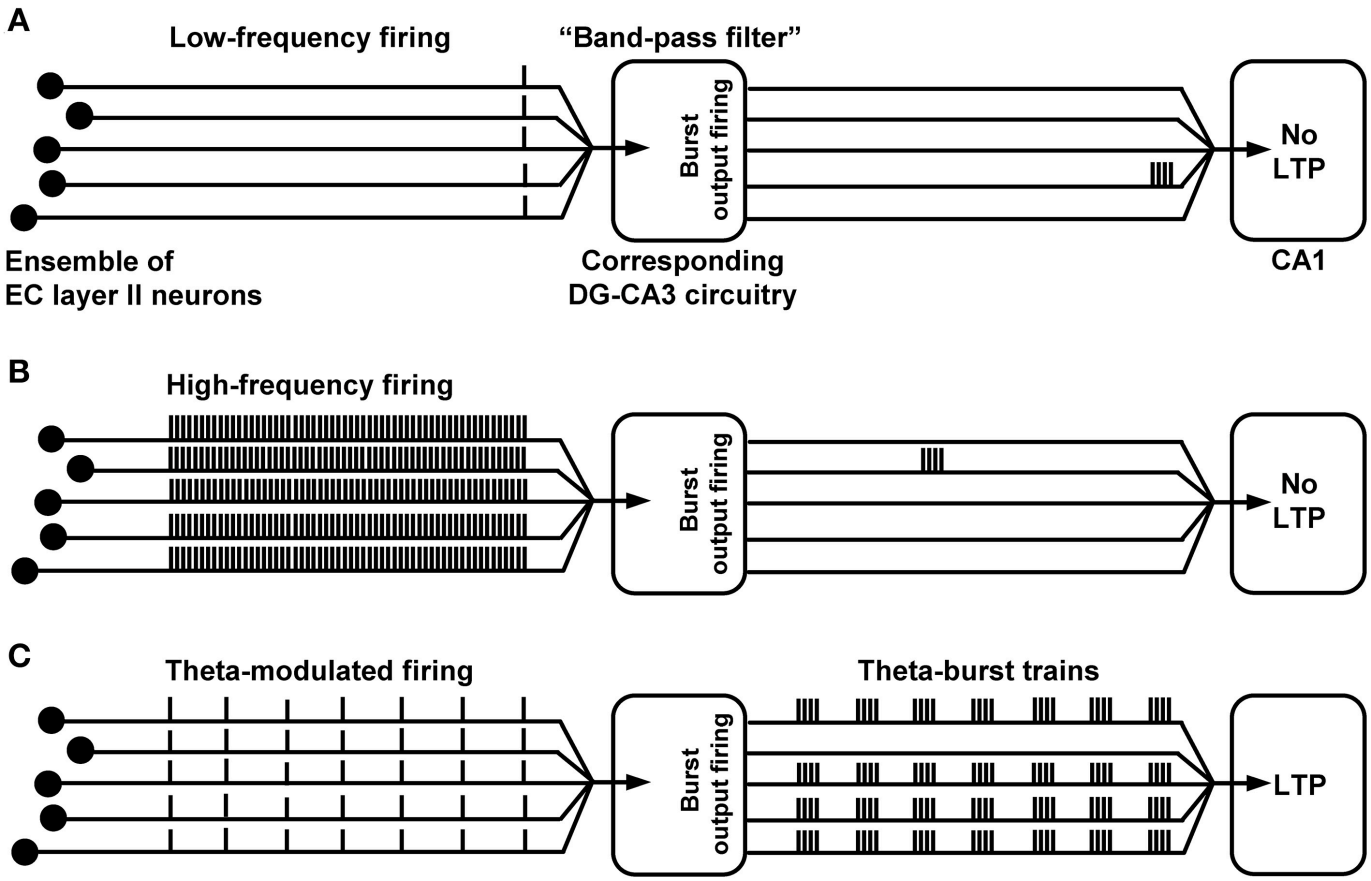
**Proposed (simplified) trisynaptic circuit dynamics which cause or fail to provoke induction of CA1 LTP. (A,B)** Due to “band-pass filter” properties of the DG-CA3 complex, low- or high-frequency spike activity in an EC layer II cell ensemble (even if synchronized) produces only marginal burst firing of CA3 pyramidal neurons and, thus, fails to cause induction of CA1 LTP. **(C)** Theta-modulated discharge activity in the same EC ensemble provokes synchronized theta-burst spiking of CA3 pyramidal cells, leading to LTP induction at CA3-CA1 synapses.

As stated in the introduction, much evidence speaks in favor for an essential role of CA1 LTP in the formation of some explicit memories in mammals. If so, induction of CA1 LTP should be triggered by sensory information transfer to the hippocampus. How could this take place at the level of the trisynaptic circuit network? We propose that synchronous theta-rhythmical spiking in EC layer II cell ensembles, which occurs during EC theta oscillations (Mizuseki et al., 2009; Quilichini et al., 2010; Burgalossi et al., 2011), can be an effective starting process. Indeed, we found that trisynaptic circuit waves induced by 5 Hz EC/DG-input involve high-frequency firing (>100 Hz) of CA3 pyramidal neurons and cause induction of NMDA receptor-dependent CA1 LTP within a few seconds (Stepan et al., 2012). Such trisynaptic circuit waves precisely follow the input rhythm, indicating that the activated DG granule cells also discharge in a theta-rhythmical manner. Consistently, EC theta oscillations are tightly associated with theta-modulated spiking of DG granule cells (Jung and McNaughton, 1993; Skaggs et al., 1996; Mizuseki et al., 2009; Pernía-Andrade and Jonas, 2014). The resultant frequency facilitation/depression at mossy fiber synapses (see above) causes firing of CA3 pyramidal neurons, which typically respond with burst spiking (100–300 Hz, ~4–6 action potentials per burst) to suprathreshold depolarizations (Andersen et al., 2007). Pharmacologically induced burst discharges in CA3 pyramidal neurons as well as theta-burst stimulation of CA3-CA1 projections efficiently evoke CA1 LTP (Buzsáki et al., 1987; Bliss and Collingridge, 1993). A simplified scheme of the described circuit process and trisynaptic circuit dynamics, which presumably fail to cause induction of CA1 LTP, is shown in Figure 3.

The remarks given above suggest that trisynaptic circuit waves and induction of CA1 LTP by them also occur naturally at the level of sparse numbers of trisynaptic interconnections and associated microcircuits (Jung and McNaughton, 1993; Whitlock et al., 2006; Leutgeb et al., 2007). This does not exclude contributions of other excitatory inputs (e.g., direct EC inputs to CA areas) and intrahippocampal pathways (e.g., commissural-CA3 pathway, Hagena and Manahan-Vaughan, 2011) to induction of CA1 LTP, but ascribes a major role to EC/DG-input. This might be the reason why some forms of hippocampus-dependent learning need the integrity of the entire trisynaptic circuit (Nakashiba et al., 2008) and are impaired if mossy fiber to CA3 neurotransmission is inhibited (Daumas et al., 2009).

Prior to our work, “polysynaptic LTP” in area CA1 has already been described by Buzsáki (1988) and Nakagami et al. (1997). However, there are strong differences to our study. Buzsaki induced population spike LTP by 400 Hz stimulation of the angular bundle. It is questionable if 400 Hz firing is a physiological activity pattern of EC layer II cells (Andersen et al., 2007) and Buzsáki states that direct EC inputs to CA areas, which we intentionally eliminated in our experiments (Figure 1A), were presumably essential for the population spike LTP. Moreover, he points out that trisynaptic interconnections are not able to follow high-frequency activity in the perforant path. In the second study, Nakagami and colleagues performed VSDI recordings in hippocampal slices and claimed that 100 Hz stimulation of the DG's dendritic field provoked trisynaptic LTP induction. Yet, the stimulation paradigm employed most likely did not only trigger neurotransmission at perforant path-DG granule cell synapses, but also led to direct perforant path inputs to CA3 pyramidal neurons and non-synaptic excitation of DG granule cells. Furthermore, in this study no clear evidence for the occurrence of CA1 LTP is provided, e.g., by blocking the LTP via NMDA receptor inhibition.

Finally, it is important to mention that LTP in the hippocampus has been evidenced to be related to hippocampal theta and gamma oscillations (Bikbaev and Manahan-Vaughan, 2008). For instance, it has been shown that high-frequency activity in the Schaffer collateral-commissural pathway induces CA1 LTP if triggered during the positive phase of the hippocampal theta rhythm. In contrast, if evoked during the negative phase, long-term depression (LTD) occurs (Hölscher et al., 1997; Hyman et al., 2003). Our VSDI assay does currently not allow investigations on such relationships for plasticity induction by trisynaptic circuit waves. Anyhow, this assay revealed a circuit process that illustrates how sensory information transfer from the EC to the DG can produce high-frequency activation of CA3-CA1 synapses.

## Summary

Based on our recent work (Stepan et al., 2012) and studies from other groups, we developed a neurophysiological scenario illustrating how sensory information transfer from the EC to the hippocampus might cause induction of memory-associated CA1 LTP. We describe data suggesting that theta-frequency EC/DG-input (generated by theta-modulated spiking in EC layer II cell ensembles) can effectively overcome “band-pass filter” mechanisms of the DG-CA3 complex, producing activity waves which propagate through the entire trisynaptic circuit network. Such trisynaptic circuit waves start to appear in an initially progressive manner a few hundred milliseconds after the onset of EC/DG-input and involve high-frequency firing of CA3 pyramidal neurons, leading to a rapid induction of NMDA receptor-dependent CA1 LTP. Therefore, an important physiological function of EC theta oscillations might be to drive sensory information through the whole trisynaptic entorhinal-hippocampal loop and to “buffer” it in area CA1. These processes, however, only take place if EC/DG-input exhibits a certain amount of persistence (>for CA1 LTP induction), which is reminiscent of the observation that episodic and spatial learning typically needs a certain time of content exposure.

## Outlook: “all optical” probing of hippocampal network dynamics *in vitro*

A comprehensive elucidation of the mechanisms and physiological functions of hippocampal network dynamics does not only require sophisticated recording methods like fast VSDI or multi-electrode array techniques, but also tools which allow a temporally precise activation or silencing of specific types of neurons. The latter is now provided by the optogenetic toolbox (e.g., Zhang et al., 2010) and a combination with VSDI has already been proposed to yield an useful “all optical” approach for studying brain circuit dynamics *in vitro* (Airan et al., 2007a). Due to the data we obtained by VSDI (Refojo et al., 2011; von Wolff et al., 2011; Stepan et al., 2012; Avrabos et al., 2013), we also consider this approach promising. Especially its refinement by genetically encoded voltage indicators, which supersede staining of the neuronal network under study with a conventional VSDI dye and enable one to optically record from specific cell types (Flytzanis et al., 2014) appears valuable for future research. This also applies to two-photon activation of optogenetically used opsins (Prakash et al., 2012).

### Conflict of interest statement

The authors declare that the research was conducted in the absence of any commercial or financial relationships that could be construed as a potential conflict of interest.
